# Complete mitochondrial genome of *Gynaikothrips ficorum* (Thysanoptera: Phlaeothripidae)

**DOI:** 10.1080/23802359.2021.1923412

**Published:** 2021-06-21

**Authors:** Lihong Dang, Xia Wang, Danle Xie, Yuxin Gao, Linpeng Zhao

**Affiliations:** a School of Biological Science & Engineering, Shaanxi University of Technology, Hanzhong, China; b Shaanxi Changqing National Nature Reserve, Hanzhong, China

**Keywords:** Thysanoptera, mitochondrial genome, phylogeny

## Abstract

*Gynaikothrips ficorum* (Marchal 1908) is a major pest of bonsai ficus and poses a considerable economic threat to gardening industry. The mitochondrial genome of *G. ficorum* was sequenced and annotated in this study. Its whole mitogenome was 15,313 bp in length, including 37 typical genes in animal mitogenomes. ATN was used as start codon in most of the PCGs except for *nad4l*, which used TTG. All PCGs used TAA as termination codon except *atp8* and *atp6* which were ended with an incomplete T and TAG, respectively. A phylogenetic tree based on complete mitochondrial genomes of 17 species (15 Thysanoptera species and two outgroups) showed that the monophyly of Phlaeothripidae was supported and *G. ficorum* and *G. uzeli* formed a sister group.

A major pest of bonsai ficus, *Gynaikothrips ficorum* (Marchal 1908) preferentially feeds on tender young leaves and induces leaf-fold galls on *Ficus microcarpa* (Okajima 2006). The infested trees will not be killed, but the ornamental value and quality of the plants are reduced markedly (Han 1997). In this study, the samples of *G*. *ficorum* were collected from *F. microcarpa* located beside the road at Yibin City in Sichuan Province, China (104°60′04″E, 28°75′14″N) in 2015. Fresh specimens were preserved in 95% ethanol for slide mounting and DNA extraction. Voucher specimens (JM2015051) were identified by morphology, and deposited in Qinba Biological Museum, Shaanxi University of Technology, Hanzhong, China (QBM). The genomic DNA extraction of *G. ficorum* is used by the DNeasy kit (Qiagen, Hilden, Germany). The paired end libraries were constructed and sequenced (2 × 150bp) using the Illumina HiSeq 4000 platform by Nextomics Bioscience Company (Wuhan, China). The complete mitogenome for this species was assembled and annotated by MITOZ (Meng et al. 2019).

The complete mitogenome of *G. ficorum* is a typical circular double-stranded DNA molecule of 15,313 bp in length (GenBank accession no. MT892761). It consists of 13 protein-coding genes (PCGs), 22 transfer RNA genes in which *trnM* and *trnY* are duplicated, two ribosomal RNA genes and one control region (d-loop). Twenty-four genes (4 PCGs, 18 tRNA and 2 rRNA genes) are transcribed on the majority strand (J-strand), with the remaining genes being located on the minority strand (N-strand). The overall nucleotide composition of the *G. ficorum* mitogenome is 42.8% T, 8.6% C, 40.3% A and 8.3% G, with a strong bias toward A + T (83%), AT- and GC- skew of the whole mitogenome of *G. ficorum* is −0.029 and −0.017, respectively. Most PCGs start with ATN codon, expect *nad4l*, which starts with TTG codon. Most PCGs were ended with TAA codon expect *atp8* and *atp6* which are ended with an incomplete T and TAG, respectively. A single putative control region, rather than two or more in some other thrips species (Yan et al. 2012; Shao and Baker 2003; Yan et al. 2014; Dickey et al. 2015; Chakraborty et al. 2018; Kumar et al. 2019), is found in the mt genome of *G. ficorum*. The unique control region is 251 bp in length and located between *nad5* and *trnF*. The secondary structure of tRNA were predicted by MITOS (Bernt et al. 2013) and found that except *trnS1* loses a dihydrouridine (DHU) arm, all others form a typical clover-leaf structure.

Comparing the arranged mitochondrial genes of *G. ficorum* with inferred insect ancestral arrangement, we found severe rearrangements in the *G. ficorum* mitochondrial genome. Most genes arranged irregularly but two gene clusters (*atp8-atp6-cox3* and *nad4-nad4L*) conserved. The mt genes of this target species arranged in *cox1-trnQ-cox2-atp8-atp6-cox3-nad3-trnN-trnS1-trnE-nad5-trnF-trnH -trnS-trnC-nad4-nad4L-trnT-nad6-cytb-trnR-trnA-trnY2-rrnS-trnD-trnG-trnL1-trnW-trnM1-trnY1-trnM2-trnV-trnK-trnI-nad2-trnL2-rrnL-trnP-nad1*.

A phylogenetic tree was constructed based on the complete mitochondrial genomes of 15 Thysanoptera species together with two species *Aphis gossypii* and *Alloeorhynchus bakeri* as outgroups using the maximum-likelihood (ML) method with 1000 bootstrap replicates by MEGA7.0 (Sudhir et al. 2016) (Figure 1). The phylogenetic relationships indicated by the tree supported completely their taxonomic relationships based on morphological characteristic (ThripsWiki 2020), in which *G. ficorum* and *G. uzeli* were clustered into a clade, and they formed a sister clade to *H. aculeatus*. The result showed the three species belonging to two genera have close relationships, and it supported the monophyly of Phlaeothripidae.

**Figure 1.**
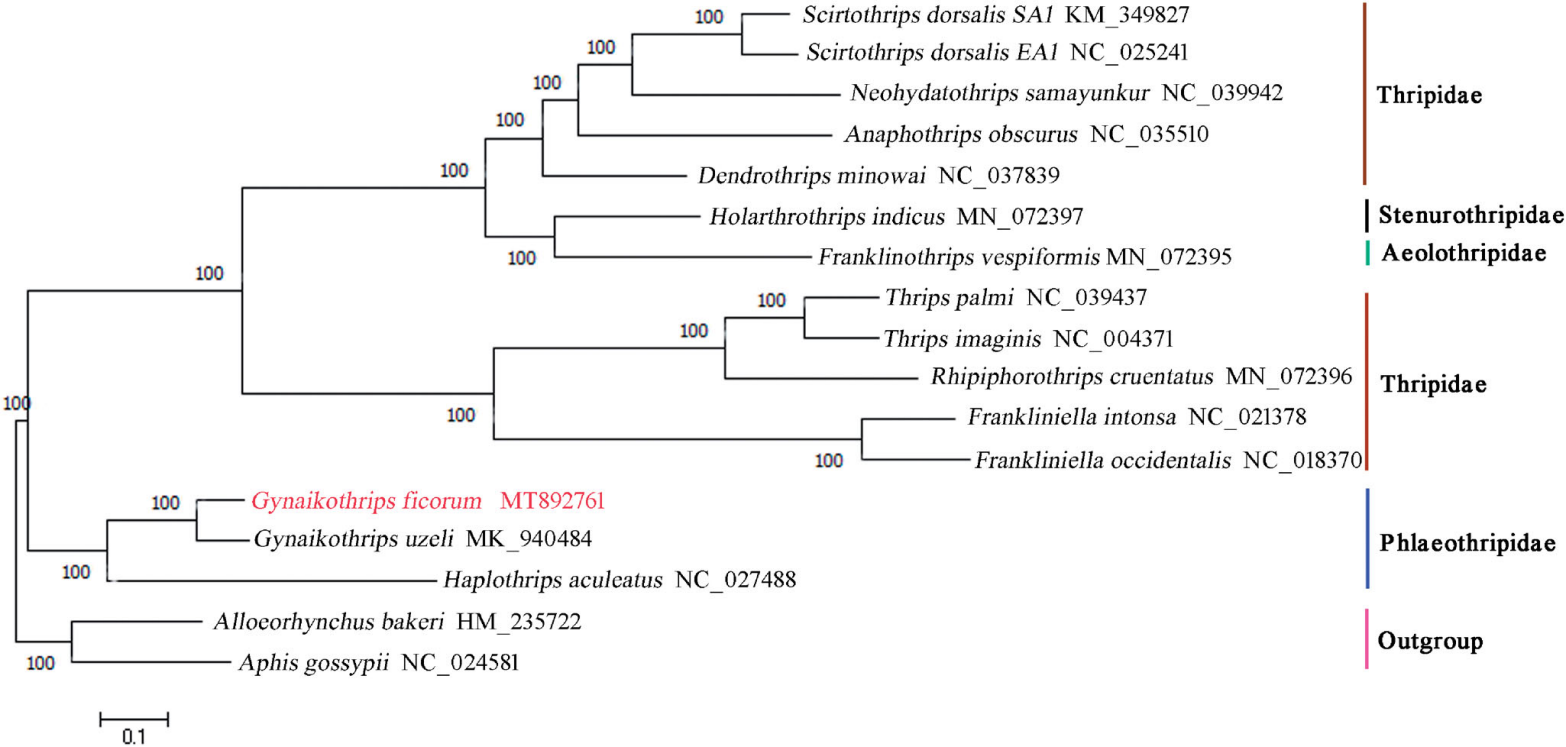
The maximum-likelihood (ML) phylogenetic tree of Thysanoptera.

## Data Availability

The data that support the findings of this study are openly available in GenBank at https://www.ncbi.nlm.nih.gov/genbank/, reference number MT892761. The associated SRA, BioProject and Bio-Sample numbers are as follows: SRR14205964, PRJNA721109, and SAMN18700104.
